# Buruli Ulcer (M. ulcerans Infection): New Insights, New Hope for Disease Control

**DOI:** 10.1371/journal.pmed.0020108

**Published:** 2005-04-26

**Authors:** Paul D. R Johnson, Timothy Stinear, Pamela L. C Small, Gerd Pluschke, Richard W Merritt, Francoise Portaels, Kris Huygen, John A Hayman, Kingsley Asiedu

## Abstract

Buruli ulcer is a disease of skin and soft tissue caused by *Mycobacterium ulcerans.* It can leave affected people scarred and disabled. What are the prospects for disease control?

Buruli ulcer is a disease of skin and soft tissue with the potential to leave sufferers scarred and disabled. It is caused by an environmental pathogen, Mycobacterium ulcerans, that produces a destructive toxin. The exact mode of transmission is unclear. The main burden of disease falls on children living in sub-Saharan Africa, but healthy people of all ages, races, and socioeconomic classes are susceptible.

## History and Epidemiology

M. ulcerans is the third most common mycobacterial pathogen of humans, after M. tuberculosis and M. leprae (which cause tuberculosis and leprosy, respectively). The definitive description of M. ulcerans was published in 1948, when MacCallum and others in Australia reported six cases of an unusual skin infection caused by a mycobacterium that could only be cultured when the incubation temperature was set lower than for M. tuberculosis [1]. In Africa, large ulcers almost certainly caused by M. ulcerans had been described by Sir Albert Cook in 1897 and by Kleinschmidt in northeast Congo during the 1920s [2].

The main burden of disease falls on children living in sub-Saharan Africa.

Prior to the 1980s, foci of M. ulcerans infection were reported in several countries in sub-Saharan Africa including Congo [3], Uganda [4], Gabon, Nigeria [5], Cameroon, and Ghana [6]. The Uganda Buruli Group coined the name “Buruli ulcer” because the cases they described were first detected in Buruli county, near lake Kyoga [7].

**Figure 1:**
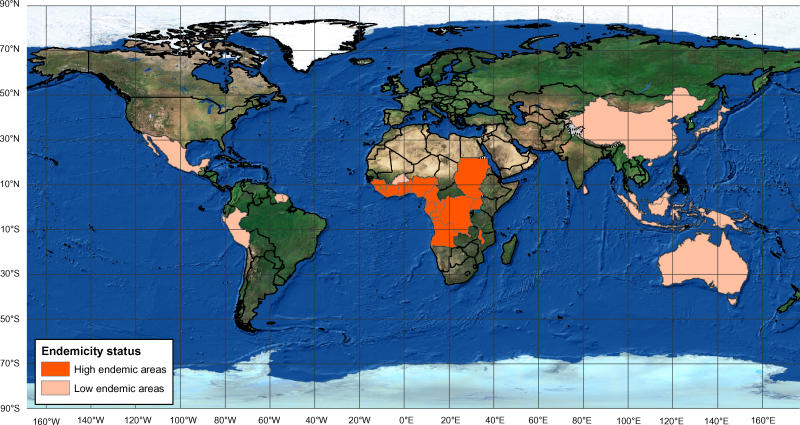
Countries Reporting Buruli Ulcer The boundaries and names shown and the designations used on this map do not imply the expression of any opinion whatsoever on the part of the World Health Organization concerning the legal status of any country, territory, city, or area or of its authorities, or concerning the delimitation of its frontiers or boundaries. Dotted lines on maps represent approximate border lines for which there may not yet be full agreement. Data Source: WHO/Global Buruli Ulcer Initiative Map Production: Public Health Mapping & GIS Communicable Diseases (CDS) World Health Organization. (Photo: World Health Organization)

Since 1980, dramatic increases in the incidence of Buruli ulcer have been reported from the West African countries of Benin [8], Côte d'Ivoire [9], and Ghana [10]. New foci were also discovered recently in Togo [11] and Angola [12]. A characteristic of Buruli ulcer is its focal distribution even within endemic regions, and obtaining accurate disease burden estimates is difficult. However, in some highly endemic districts in Ghana, point prevalence has been estimated to be as high as 150.8/100,000 individuals [10], and in southern Benin, a recent study has reported detection rates of 21.5/100,000 per year, higher than for either tuberculosis or leprosy [8]. In West Africa, about 25% of people affected by the disease, mostly children, are left with permanent disabilities. The disease is also endemic in several other countries outside Africa, including rural areas of Papua New Guinea, Malaysia, French Guiana, and Mexico (Figure 1). In Australia, the disease remains uncommon, but there have been increases in both incidence and the number of endemic areas in the last 15 years [13,14].

## Causative Organism and Pathology

Mycobacterium ulcerans is a slow-growing environmental mycobacterium that can be cultured from human lesions on mycobacterial medium at 30–32 °C [15]. Histological specimens typically show large clumps of extracellular acid-fast organisms surrounded by areas of necrosis and a poor or absent inflammatory response [16].

Subcutaneous fat is particularly affected, but underlying bone may also become involved in advanced cases [15]. The pathogenesis and histological appearance is explained by a recently identified diffusible lipid toxin, mycolactone [17]. Later in the natural history of the disease, the immunosuppressive effect of the toxin is somehow overcome by the host, immunity develops, and healing commences.

## Clinical Features

**Figure 2:**
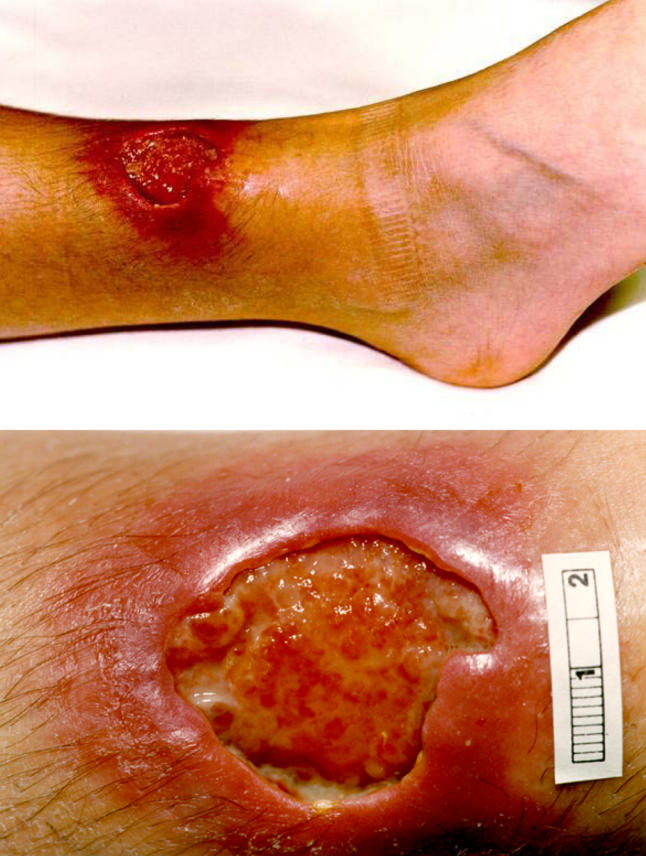
M. ulcerans Infection of the Shin of an 11-Year-Old Boy, Coastal Victoria, Australia A closer view, revealing deep undermining, is shown in the second panel. (Photo: Paul D. R. Johnson)

The classic lesion is a necrotic skin ulcer with deeply undermined edges (Figure 2). Any part of the body can be affected, but most lesions occur on limbs. The ulcers are slowly progressive and usually painless, and the patient is usually systemically well, which may explain why sufferers often delay seeking medical assistance. Early Buruli lesions may initially appear as a mobile subcutaneous nodule, a papule, or a raised plaque.

A subgroup of patients present with rapidly progressive oedema of a whole limb, abdominal wall, or side of the face without an obvious focal lesion. Part or all of the affected area will subsequently ulcerate, although anecdotal reports suggest that timely antibiotic therapy may greatly reduce the resulting necrosis [18].

## Treatment

The aim of treatment is to halt the infection and repair existing damage. Large ulcers are usually treated surgically to remove necrotic tissue and to graft the resulting defect. Relapse after surgery may occur in 18%–47% of cases [19], so surgeons commonly ensure wide excision margins in the hope of curing the infection.

Traditionally, drug therapy has been considered ineffective, but recent data suggest that combinations of anti-mycobacterial antibiotics that include rifampicin and either streptomycin or amikacin are able to kill M. ulcerans in human lesions [14,20]. Provisional guidelines now recommend the use of selected anti-mycobacterial drugs, usually combined with surgery, for the treatment of Buruli ulcer [21].

## Prevention of Disabilities

**Figure 3:**
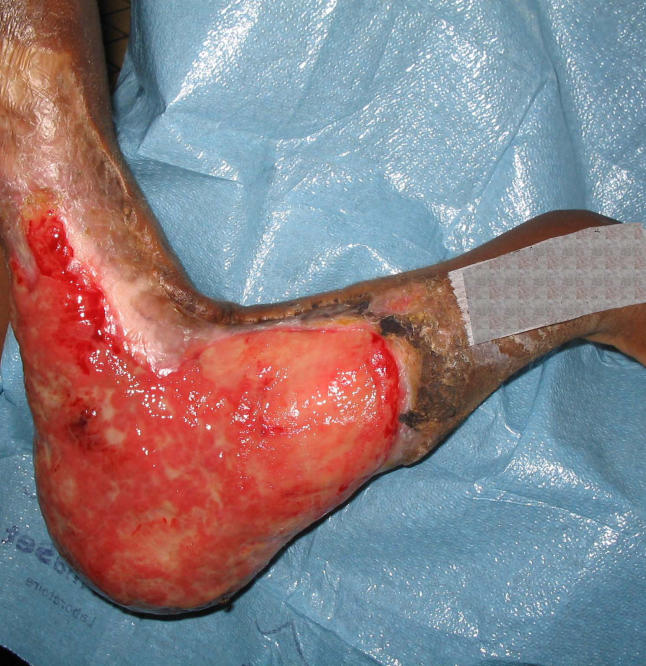
Long-Term Sequelae of M. ulcerans Infection, Benin, West Africa (Photo: Kingsley Asiedu)

Untreated Buruli ulcer will eventually subside with the gradual development of host immunity in most cases. However, by this time, tissue damage may be very extensive and healing by scar can lead to permanent functional and cosmetic deformity (Figure 3). Successful treatment will shorten the course of the disease and minimise deformity. Skilled surgery, expert post-operative nursing care, and restorative physiotherapy are often required to achieve good outcomes. The cost of this may be beyond the means of local rural health services. Even in Australia, where there is universal access to health care, the cost and complexity of treating M. ulcerans infections can be considerable [22].

## Public Health Efforts

Case control studies have suggested that farming activities close to rivers in endemic areas are a risk factor for Buruli ulcer [9], but for farmers involved in subsistence agriculture, avoidance of riverine areas is difficult. A recent study from Ghana has suggested that swimming in rivers may also be an independent risk factor [23]. To date, the main focus of public health efforts has been on early detection and treatment, which greatly reduce morbidity and cost [8,24].

## Why Has Buruli Ulcer Been Neglected until Now?

Despite its long history, Buruli ulcer has gone largely unnoticed until recently. Buruli ulcer typically occurs in poor rural communities with little economic or political influence. Rural isolation may mean that national surveillance systems do not immediately detect the appearance of new outbreaks. Affected populations may believe that there is no effective medical treatment for the disease, which discourages them from seeking assistance [25,26].

In the developed world, Buruli ulcer is frequently omitted from standard medical texts and undergraduate medical courses. The absence of a potentially profitable market has meant that there has been little private investment to date in drug and vaccine development or in research to improve prospects for better control.

## Future Directions and the End of Obscurity

### Global Buruli Ulcer Initiative.

In December 1997, Hiroshi Nakajima, then Director-General of the World Health Organization (WHO), announced that WHO would take the lead to mobilise the world's expertise and resources to fight the emergence of Buruli ulcer as a serious public health problem. In 1998, WHO launched the Global Buruli Ulcer Initiative to coordinate control and research efforts, and organised the first International Conference on Buruli ulcer control and research in Yamoussoukro, Côte d'Ivoire. The resulting “Yamoussoukro Declaration on Buruli Ulcer” drew attention to the severity of the disease as an emerging public health problem and expressed concern about its many poorly understood features. In May 2004, the World Health Assembly adopted a resolution on Buruli ulcer that called for increasing surveillance and control, and for intensified research to develop tools to diagnose, treat, and prevent the disease [27]. The attention of the affected countries, donor agencies, nongovernmental organisations, and the research community has been captured by these and other initiatives, and we are now entering an exciting period of rapidly expanding knowledge and interest in the disease. These developments will ensure that Buruli ulcer is not neglected again.

### Recent research on transmission.

**Figure 4:**
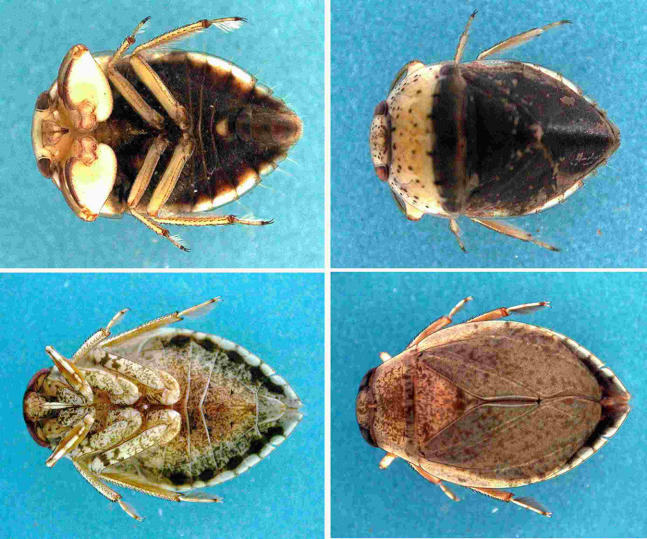
Semi-Aquatic Hemiptera That Have Tested Positive for M. ulcerans The top row is Macrocoris sp., 1.0 centimeter in body length (Family Naucoridae), and the bottom row is Appasus sp., about 2.5 centimeters in body length (Family Belastomatidae). The ventral and dorsal views are in the left and right panels, respectively.

For over 50 years we have known that proximity to marshes and wetlands, often created as a result of some human environmental disturbance, is a risk factor for infection [28], but the exact mode of transmission remains an enigma. M. ulcerans was first detected in the environment in the 1990s by Australian researchers using polymerase chain reaction (PCR) [29,30]. Subsequently, PCR was used by others to identify M. ulcerans in aquatic insects obtained from endemic areas in Africa [31], leading to the hypothesis that M. ulcerans may be transmitted by biting water bugs of the insect order Hemiptera (Naucoridae and Belostomatidae; Figure 4).

In support of this hypothesis, M. ulcerans has been detected in the salivary glands of Naucoris sp., and has been transmitted to laboratory mice via this aquatic insect [32,33]. There is additional evidence that M. ulcerans DNA can be detected by PCR in other aquatic insect predators (e.g., Odonata and Coleoptera), as well as in aquatic snails, small fish, and the biofilm of aquatic plants [34]. Despite this, only two pure cultures of M. ulcerans have been obtained from environmental sources. In Australia, it has been postulated that aerosols arising from contaminated water may disseminate M. ulcerans and infect humans via the respiratory tract, or through contamination of skin lesions and minor abrasions [35,36], but this has yet to be proven. Recent progress has been rapid, but the exact mode of transmission, the key important reservoir species, and transmission of M. ulcerans through the aquatic food chain remain to be elucidated.

### Immune response.

The immune mechanisms involved in protection against Buruli ulcer are also largely unknown at present. Interestingly, peripheral blood mononuclear cells obtained from people with a past or current M. ulcerans infection typically show a strong T helper (Th)–2 cytokine response when exposed in vitro to M. ulcerans. In contrast, samples obtained from their household contacts (exposed healthy controls) exhibit a Th-1 immune response, suggesting that natural resistance may be determined by cell-mediated immune mechanisms directed against intracellular organisms [37]. In one fascinating case study, it has been shown that the development of ulcerative M. ulcerans disease is associated with a shift from the Th-1 to the Th-2 phenotype [38]. Interleukin-10 may be a key cytokine that mediates local Th phenotype switching within nodules and ulcers [39].

Antibodies may also have a protective role against M. ulcerans, as the pathogen is extracellular during active disease. Experimental infection of mice genetically inactivated in various compartments of the immune response (B lymphocytes, Th cells, and cytolytic T lymphocytes, cytokines, and monokines) will help us to understand how host immunity is acquired.

### Developing new drugs.

M. ulcerans is susceptible to several anti-mycobacterial drugs in vitro, but the most promising results in the mouse footpad model were obtained with a combination of rifampicin and amikacin [40]. A human trial has recently shown that early nodular lesions may be rendered culture-negative after a minimum of four weeks therapy with rifampicin plus streptomycin [14,20].

Further research to identify cheap, safe, and effective oral combinations that can be used as an adjuvant to surgery or that could even replace surgery for early lesions is urgently required. At least one new compound, which appears safe for humans in early phase I trials, has remarkable activity in vitro against many mycobacterial species including M. tuberculosis and M. ulcerans [41].

### M. ulcerans toxin.

M. ulcerans makes a family of toxic macrolides, the mycolactones, that are required for virulence [17]. Mycolactone causes cells in cell culture assays to undergo apoptosis and necrosis and produces a lesion that closely resembles Buruli ulcer when injected directly into guinea pig skin [42]. Although toxic lipid molecules are relatively commonly produced by mycobacteria, the synthesis of mycolactone itself appears to be restricted to M. ulcerans [43].

### Prospects for developing a vaccine.

There is no specific vaccine against M. ulcerans, but the M. bovis BCG vaccine offers some protection, albeit short lived [44,45]. BCG may possibly provide more enduring protection against the most severe forms of Buruli ulcer [46].

Current prospects for better vaccines include improved or repeated BCG vaccination, rational attenuation of a live M. ulcerans isolate, or subunit vaccines aimed at protein antigens or the toxin mycolactone itself [47].

### Genome, bacterial population structure, and serodiagnosis.

The expected publication of the whole M. ulcerans genome sequence in 2005 will mark a major milestone for Buruli ulcer researchers. Already the project has uncovered the presence of a large virulence plasmid that encodes mycolactone production proteins [48]. Biosynthesis of mycolactone requires three polyketide synthase enzymes and at least two accessory enzymes, all of which are located within a 110-kb cluster on this plasmid. The genome project has also revealed a remarkably high copy number of two insertion sequences, accounting for more than 5% of the total genome. There is evidence of considerable genome decay, with many potential pseudogenes and DNA deletions. These data coincide with accumulating evidence that suggests a reservoir in insects or other aquatic species and indicate that M. ulcerans may be passing through an evolutionary bottleneck as it adapts to life in a specialised niche environment.

Molecular typing of M. ulcerans isolates has revealed a clonal population structure within specific geographical regions. Innovative genetic fingerprinting methods will be required to reveal local transmission pathways and environmental reservoirs.

In endemic areas, clinical diagnosis of advanced Buruli ulcer lesions by experienced clinicians is quite reliable. Diagnostic confidence can be increased by detecting acid-fast bacilli in smears or biopsies, and the diagnosis confirmed by culture or PCR [49]. A recent report describes a new dry-reagent PCR for Buruli ulcer that could be used in small regional centres, and reports from Ghana suggest a sensitivity of 95% [50]. Diagnosis of Buruli ulcer outside endemic areas or of pre-ulcerative lesions can be challenging. The genome sequence will greatly assist the development of a noninvasive serodiagnostic assay based on M. ulcerans–specific antigens.

## Conclusion

Buruli ulcer is now emerging from long years of neglect: interest and momentum are growing. However, there is much to do if we are to understand why the disease is becoming more common and how this relates to human activity. The current control strategy of early detection and treatment should be scaled up in the affected countries. Our ultimate goal is the development of an effective and safe vaccine able to provide long-lasting protection for those who live in endemic areas.

## Abbreviations

PCR: polymerase chain reaction
Th: T helper
WHO: World Health Organization
