# Correction: Buruli Ulcer (*M. ulcerans* Infection): New Insights, New Hope for Disease Control

**DOI:** 10.1371/journal.pmed.0020173

**Published:** 2005-05-31

**Authors:** Paul D. R Johnson, Timothy Stinear, Pamela L. C Small, Gerd Pluschke, Richard W Merritt, Francoise Portaels, Kris Huygen, John A Hayman, Kingsley Asiedu

In PLoS Medicine, vol 2, issue 4, DOI: 10.1371/journal.pmed.0020108

In the PDF versions of the article, the fourth author's name should be spelled Gerd Pluschke in the list of authors and the citation.

